# Silencing of the Nicotiana benthamiana phytoendesaturase gene by root treatment of exogenous dsRNA

**DOI:** 10.18699/vjgb-25-123

**Published:** 2025-12

**Authors:** Т.S. Golubeva, V.A. Cherenko, E.A. Filipenko, I.V. Zhirnov, A.A. Ivanov, A.V. Kochetov

**Affiliations:** Institute of Cytology and Genetics of the Siberian Branch of the Russian Academy of Sciences, Novosibirsk, Russia Immanuel Kant Baltic Federal University, Kaliningrad, Russia; Institute of Cytology and Genetics of the Siberian Branch of the Russian Academy of Sciences, Novosibirsk, Russia; Institute of Cytology and Genetics of the Siberian Branch of the Russian Academy of Sciences, Novosibirsk, Russia; Institute of Cytology and Genetics of the Siberian Branch of the Russian Academy of Sciences, Novosibirsk, Russia; Institute of Cytology and Genetics of the Siberian Branch of the Russian Academy of Sciences, Novosibirsk, Russia Novosibirsk State University, Novosibirsk, Russia; Institute of Cytology and Genetics of the Siberian Branch of the Russian Academy of Sciences, Novosibirsk, Russia

**Keywords:** RNA interference, gene silencing, phytoene desaturase, exogenous dsRNA, Nicotiana benthamiana, root treatment, РНК-интерференция, генный сайленсинг, фитоендесатураза, экзогенная дцРНК, Nicotiana benthamiana, корневая обработка

## Abstract

RNA interference (RNAi) is a powerful tool for gene silencing. It has recently been used to design promising plant protection strategies against pests such as viruses, insects, etc. This generally requires modifying the plant genome to achieve in planta synthesis of the double-stranded RNA (dsRNA), which guides the cellular RNA interference machinery to silence the genes of interest. However, given Russian legislation, the approach in which dsRNA is synthesized by the plant itself remains unavailable for crop protection. The use of exogenously produced dsRNA appears to be a promising alternative, allowing researchers to avoid genetic modification of plants, making it possible to implement potential results in agriculture. Furthermore, exogenous dsRNAs are superior to chemical pesticides (fungicides, insecticides, etc.), which are widely used to control various plant diseases. The dsRNA acts through sequence-specific nucleic acid interactions, making it extremely selective and unlikely to harm off-target organisms. Thus, it seems promising to utilize RNAi technology for agricultural plant protection. In this case, questions arise regarding how to produce the required amounts of pathogen-specific exogenous dsRNA, and which delivery method will be optimal for providing sufficient protection. This work aims to utilize exogenous dsRNA to silence the Nicotiana benthamiana phytoene desaturase gene. Phytoene desaturase is a convenient model gene in gene silencing experiments, as its knockdown results in a distinct phenotypic manifestation, namely, leaf bleaching. The dsRNA synthesis for this work was performed in vivo in Escherichia coli cells, and the chosen delivery method was root treatment through watering, both techniques being as simple and accessible as possible. It is surmised that the proposed approach could be adapted for broader use of RNAi technologies in agricultural crop protection.

## Abstract (ru)

РНК-интерференция – мощный инструмент для генного сайленсинга, благодаря чему используется при разработке новых подходов с большим потенциалом для защиты растений от вирусов, насекомых и других патогенов. Как правило, в таких системах геном растений подвергается модификациям с целью синтеза двуцепочечных РНК (дцРНК), необходимых для РНК-интерференции и последующего сайленсинга непосредственно в растительных клетках. Однако с учетом законодательства Российской Федерации такой подход не может использоваться на сельскохозяйственных растениях, что делает невозможным его применение при условии синтеза дцРНК самим растением. Применение экзогенно синтезированной дцРНК может стать перспективным способом защиты растений, так как позволяет избежать создания генетически модифицированных организмов и внедрить полученную разработку в сельском хозяйстве. Также экзогенные дцРНК имеютпреимущество по сравнению с химикатами (фунгицидами, инсектицидами и т. д.), используемыми для защиты растений, так как дцРНК действуют посредством своей специфической нуклеотидной последовательности, что делает описанный подход крайне избирательным к патогену и безопасным для других организмов. В совокупности вышеперечисленные факторы делают методы РНК-интерференции весьма перспективными для применения в сельском хозяйстве с целью защиты растений, поэтому встает вопрос о крупномасштабном синтезе экзогенных молекул дцРНК, специфичных к определенному патогену, и выборе оптимального способа их доставки для достижения защитного эффекта. Целью настоящей работы является сайленсинг гена фитоендесатуразы табака Бентхама (Nicotiana benthamiana) с применением экзогенно синтезированной дцРНК. Ген фитоендесатуразы – очень удобная модель в экспериментах по регуляции генной активности, так как его сайленсинг сопровождается ярким фенотипическим проявлением в виде побеления листьев. Синтез дцРНК осуществляли in vivo в клетках Escherichia сoli; в качестве способа доставки выбрана корневая обработка через полив растения – максимально простые и доступные манипуляции. Предполагается, что предложенный подход может быть масштабирован и адаптирован для защиты растений в сельском хозяйстве с помощью методов, в основе которых лежит РНК-интерференция.

## Introduction

In order to protect plants against pathogens and pests, agriculture relies on the widespread use of chemical pesticides that are applied to the environment in large amounts. These intense applications of chemical pesticides pose potential risks for human health, beneficial organisms, and the environment (Niehl et al., 2018). Therefore, it is imperative that new alternative methods of controlling plant diseases be developed. Thus, there is a need for novel tools and alternative methods to control disease epidemics. A promising new approach with strong potential for protecting plants against viruses and other pathogens involves the application of dsRNA.

dsRNA applications can be highly advantageous over chemical compounds. Whereas chemical compounds act by a structure-dependent mechanism, dsRNAs act through their specific nucleotide sequence. Hence, once engineered to affect a specific pathogen target with a homologous sequence, dsRNAs and small interfering RNA (siRNA) derivatives should act only against the targeted pathogen. It is worth noting that, unlike chemical pesticides, dsRNA agents are biocompatible and biodegradable compounds, natural and universally found inside and outside organisms as well as in food. In this way, applying dsRNA proves to be a much more flexible and environmentally friendly approach.

However, the wide application of dsRNA treatment in agriculture is hampered by the lack of efficient and costeffective methods to synthesize large quantities of dsRNA. The main approach to obtain dsRNA has been the physical annealing of two enzymatically synthesized single-stranded RNAs (ssRNA) in vitro (Laurila et al., 2002). Also, the efficiency of exogenously administered dsRNA in plants can be influenced by several factors: dsRNA concentration/dose and length/size, method of application, method of delivery, plant organ-specific activity, and stability under unfavorable environmental conditions. It is these factors that eventually determine the rate of uptake of exogenous dsRNA by plant cells for RNAi triggering. Given all these factors, it is necessary to develop an efficient method for the large-scale synthesis of dsRNA molecules and to choose the best method for their delivery (Carthew, Sontheimer, 2009).

In this work, we suggest a method for regulating the activity of the phytoene desaturase gene in tobacco (Nicotiana benthamiana) using dsRNA synthesized in Escherichia coli cells: root treatment of plants with crude bacterial lysate containing the target dsRNA led to photobleaching of plant leaves by RNA interference (RNAi).

Phytoene desaturase is a key enzyme in chlorophyll synthesis, with its silencing known to result in the phenotypic manifestation of leaf photobleaching. Thus, this gene is often used as a model for developing new approaches to regulate gene activity. Root treatment with crude lysate allows scaling up dsRNA production and minimizing delivery time and resources. The approach developed is expected to be used to protect agricultural plants by regulating gene activity by RNAi.

## Materials and methods

Characterization of the bacterial strain. This study used the E. coli HT115 (DE3) strain [F-, mcrA, mcrB, IN(rrnD-rrnE)1, rnc14::Tn10(DE3 lysogen: lacUV5 promoter – T7 polymerase) (IPTG-inducible T7 polymerase) (RNAse III minus)]. This strain is deficient in RNase III and therefore can be used to generate dsRNA. The strain was cultured in standard Luria-Bertani (LB) liquid media or LB agar with tetracycline (12.5 μg/mL).

Characterization of the L4440 plasmid. Plasmid L4440 (Plasmid #1654, Addgene, USA) was used in this study. The specific feature of this plasmid (i. e. the presence of two strong T7 promoters in opposite directions) makes it suitable for the production of dsRNA fragments. Also, the plasmid carries an ampicillin resistance gene.

Selection of primers. Primers were selected using the Primer-BLAST resource (https://www.ncbi.nlm.nih.gov/tools/ primer-blast/index.cgi) on the N. benthamiana mRNA matrix. The selected primer variants were tested for the presence of hairpins, self- and heterodimers using the OligoAnalyzer tool (https://eu.idtdna.com/calc/analyzer). As a result, the following primers were synthesized: forward 5′-GGCACTC AACTTTATAAACC-3′ and reverse 5′-CTTCAGTTTTCT GTCAAACCATATATGGAC-3′ (Syntol, Russia).

Production of cDNA. mRNA was isolated using the RNeasy Plant kit (Qiagen, USA) according to the manufacturer’s protocol, with the RNA sample obtained analyzed on a Bioanalyzer Instrument 2100 RNA analyzer (SB RAS Genomics Core Facility, ICBFM SB RAS). Reverse transcription was performed with the BiolabMix R01-250 kit (BiolabMix, Russia). The HS-qPCR SYBR Blue kit (BiolabMix, Russia) was used to generate fragments, and amplification was performed in a Bio-Rad IQ instrument (Bio-Rad, USA).

Cloning of the pds gene fragment. The pds gene was cloned in two steps. Initially, the gene was cloned into the pCR2.1 intermediate T-vector (Invitrogen, USA). Bacterial colonies containing the gene fragment were first selected using the blue-white screening. The final selection of clones was carried out based on the presence of the complete gene fragment in the restriction spectra after treatment with EcoRI endonuclease.

Cloning into the final vector L4440 was performed using the ligase-restriction method with the restriction endonucleases PstI and NcoI. For this purpose, the corresponding restriction sites were introduced into the pds gene fragment during its amplification (the same sites are present in the structure of the L4440 vector). Recombinant E. coli clones were selected on a selective medium containing ampicillin. To verify the presence of the pds gene fragment in the L4440 vector, PCR was performed using primers specific for the pds gene. The final presence of the pds gene fragment and its nucleotide sequence were confirmed by Sanger sequencing.

Transformation of the E. coli strain HT115 (DE3) with plasmid L4440. Competent cells were prepared according to the Nishimura protocol (Nishimura et al., 1990). For transformation, a 0.1 ml aliquot of bacterial suspension was mixed with 5 μl of L4440 plasmid dissolved in TE buffer (100 pg) and incubated on ice for 30 min. Next, the cells were placed in an incubator and maintained at 42 °C for 60 s, then incubated again on ice for 2 min and diluted 10-fold with pre-warmed to 37 °C LB broth. The suspension was incubated at 37 °C for 1 h to develop ampicillin resistance. Then 100 μl of the suspension was rubbed into LB agar with ampicillin (50 μg/ ml), and the cups were incubated at 37 °C for 24 h. The grown colonies were formed by transformed bacteria.

The presence of an integrated fragment of the pds gene was verified by PCR of selected colonies as well as by Sanger sequencing (SB RAS Genomics Core Facility, ICBFM SB RAS).

VIGS positive control. A model plant, N. benthamiana, was used in VIGS experiments. Seeds of N. benthamiana were germinated and then the seedlings were planted in plastic pots (10 cm in diameter) containing a mixture of universal potting soil (TerraVita, Russia), perlite and vermiculite (8:1:1, v/v), and cultivated in a growth chamber under continuous lighting at 24 °C.

Vector construction. The VIGS vectors pTRV1 (pYL192) and pTRV2 (pYL279) obtained from the Arabidopsis Biological Resource Center (ABRC, USA) were described previously (Liu et al., 2002; Burch-Smith et al., 2006). Total RNA was extracted from leaf tissues of Nicotiana tabacum cv. SR1 (the phytoendesaturase gene sequence in this species is identical to that in N. benthamiana) using Trizol reagent (Invitrogen, USA), and first-strand cDNA was synthesized using the RevertAid First Strand cDNA Synthesis Kit (Thermo Scientific, USA) according to the manufacturer’s instructions. The cDNA pool was used to amplify the NtPDS gene fragment sequence (GenBank accession number: AJ616742.1) by PCR using high-fidelity Phusion polymerase (New England Biolabs, USA) as per the manufacturer’s instruction. The selected primers were checked for hairpins, self-dimers, and heterodimers using the OligoAnalyzer tool. As a result, the following primers were synthesized: forward 5′-CACCGGCACTC AACTTTATAAACC-3′ and reverse 5′-CTTCAGTTTTCT GTCAAACCATATATGGAC-3′ (Syntol, Russia).

The resulting 413-bp PCR fragment was cloned into the pENTR/D-TOPO vector (Invitrogen, USA), verified by sequencing, and then recombined into the pTRV2 vector by carrying out an LR recombination reaction using the Gateway system (Invitrogen). The generated vector pTRV2::NtPDS was transformed by heat shock into the Agrobacterium tumefaciens GV2260 strain and used in VIGS experiments

The A. tumefaciens GV2260 strain, carrying the pTRV1, pTRV2, and pTRV2::NtPDS vectors, was separately inoculated into Luria-Bertani liquid media containing kanamycin (100 μg/mL) and rifampicin (25 μg/mL). The cultures were incubated overnight with shaking at 28 °C. The cells were harvested from the overnight cultures, re-suspended in the induction buffer (10 mM MES; 10 mM MgCl2; 250 μM acetosyringone; adjusted to pH 5.5 with 1 M KOH), and incubated for 6 h at room temperature in a shaker. After incubation, the cells were harvested from the induced cultures and resuspended in the infiltration buffer (10 mM MES, adjusted to pH 5.5 with 1 M KOH) with dilution to a final absorbance OD600 = 1.0

For leaf infiltration, A. tumefaciens strain GV2260 cultures containing pTRV1 and pTRV2 or pTRV2::NtPDS were mixed in a 1:1 (v/v) ratio and infiltrated into lower leaves of 21‐days‐old N. benthamiana plants using a 1-ml needleless syringe (Ratcliff et al., 2001; Liu et al., 2002). Infiltrated plants were maintained under constant light for 12 h at a 20 °C temperature for effective Agrobacterium T-DNA insertion (Brigneti et al., 2004).

Root treatment of N. benthamiana with a crude lysate of E. coli HT115 (DE3) containing dsRNA of phytoendesaturase. After inducing dsRNA synthesis, crude lysates of bacterial suspensions can be used as a source of exogenous dsRNA. Root treatments are also used to deliver target molecules for PTGS in plants (Jiang et al., 2014; Li et al., 2015; Dubrovina, Kiselev, 2019).

Crude lysates of the E. coli HT115 strain were prepared for root treatment, transformed with the L4440 plasmid with an integrated fragment of the pds gene. Bacterial lysates without plasmid, with incorporation of the native plasmid L4440, buffer, and water were used as controls.

The crude lysate was prepared in the following manner (Gan et al., 2010). An overnight culture of the bacteria was grown for 16 h with shaking at 37 °C in standard LB broth, with ampicillin at a concentration of 50 μg/mL added for strains with the L4440 plasmid insertion. The bacterial suspension was then diluted to OD595 = 0.5. Next, dsRNA synthesis was induced by adding IPTG at a final concentration of 0.6 mM, and the bacteria were incubated at 37 °C for 4 h. After the required time, the suspension was centrifuged to obtain a precipitate (1,500g, 15 min, 4 °C), which was then resuspended in ice buffer (50 mM Tris·HCl, 10 mM EDTA, pH 7.5) taken in 1/50 of the original volume. The tubes were then placed in ice and treated with ultrasound (20 kHz, 15 min). The resul ting lysate was centrifuged (9,000 rpm, 20 min, 4 °C), and the supernatant was used for plant treatments (2 ml per plant).

Four plants were used for each type of root treatment: water, Tris·HCl/EDTA buffer, E. coli HT115 without plasmid, E. coli HT115 with the L4440 plasmid, E. coli HT115 with the L4440 plasmid with an integrated pds fragment. All plants were grown in a sterile mixture of universal soil (TerraVita, Russia), perlite and vermiculite (8:1:1, v/v). The plants were treated three times a week (Monday, Wednesday, Friday) for a period of four weeks. Root treatment was carried out by watering the plants with supernatant resuspended in 2 ml of Tris-EDTA buffer

Extraction of dsRNA. 1 ml of the bacterial suspension was centrifuged for 5 min at 10,000 rcf, and the supernatant was removed. The precipitate was dissolved in 200 μl of a mixture of 1 M CH3COONH4 and 10 mM isoamyl alcohol and extracted with an equivalent volume of isopropanol:phenol:isoamyl alcohol in the ratio (25:24:1). Then it was incubated for 15 min at 65 °C and centrifuged for 15 min at 10,000 rcf. The supernatant was withdrawn and an equivalent volume of isopropanol was added, followed by incubation for 12 hours at –20 °C. After incubation, it was centrifuged for 30 min at 12,000 rcf. The liquid was removed carefully. The precipitate was washed twice with 70 % ethanol, with the precipitate resuspended in 10 μl of RNase-free water, and DNAase buffer was added. Next, the incubation was done for 30 min, followed by the addition of 20 μl of RNase A and 20 μl of DNase. Then, the incubation was performed for 1 hour at 37 °C. 100 μl of a isopropanol:phenol:isoamyl alcohol mixture in the ratio (25:24:1) was extracted. The mixture was stirred vigorously and centrifuged at 12,000 rcf for 15 min. The supernatant was removed, and the precipitate was washed with 200 μl of 70 % ethanol, dried at room temperature, and dissolved with 1× TE buffer. The presence and integrity of dsRNA were checked by gel electrophoresis in 1 % agarose gel.

## Results

**Production of exogenous dsRNA using an RNase-deficient E. coli HT115 strain transformed with the L4440 plasmid**

The E. coli HT115 (DE3) strain was used to produce dsRNA because it is deficient in RNase III, an enzyme that normally hydrolyzes dsRNA in the bacterial cell. The L4440 vector was used for the transformation. Due to having two strong T7 promoters in opposite directions, this vector can be used to produce target dsRNA fragments.

**Fig. 1.**
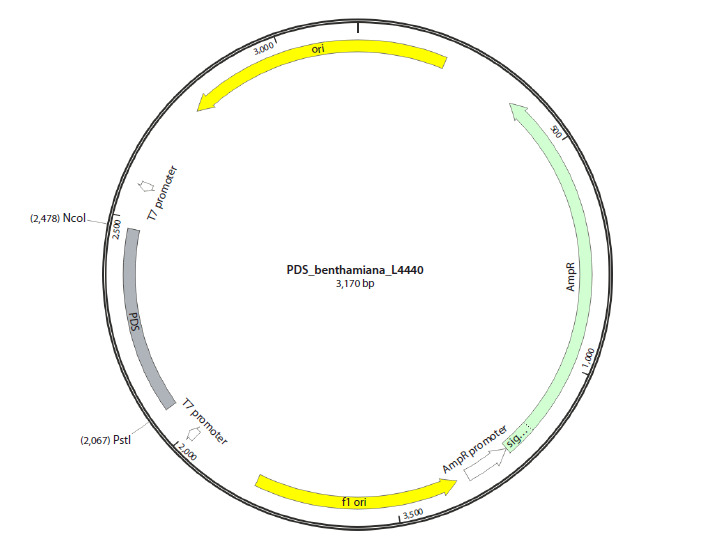
Map of vector L4440 containing a fragment of the pds gene of N. benthamiana (PDS_benthamiana_L4440).

A 404 bp fragment of the tobacco phytoene desaturase pds gene was cloned into the L4440 vector by sticky ends using restriction endonucleases PstI and NcoI, with the presence of the required fragment in the vector confirmed by sequencing. A map of the resulting plasmid is shown in Figure 1.

**Fig. 2.**
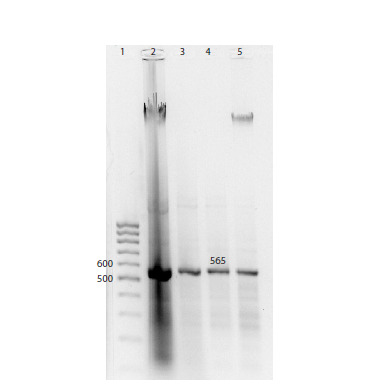
Electrophoretic analysis of dsRNA fragments synthesized in E. coli HT115 (DE3), transformed with the PDS_benthamiana_L4440 vector. 1 – 100 bp DNA marker; 2 – total nucleic acid fraction; 3 – total nucleic acid fraction treated with DNase; 4 – total nucleic acid fraction treated with RNase A and DNase; 5 – total nucleic acid fraction treated with RNase A.

The presence of the target fragments of the correct length was confirmed by electrophoretic analysis (Fig. 2).

**Preparation of a crude bacterial lysate containing dsRNA fragments for silencing phytoene desaturase N. benthamiana**

A strain in E. coli HT115 (DE3) transfected with the PDS_benthamiana_ L4440 vector was used to produce target dsRNA fragments for silencing pds genes. Induction of dsRNA synthesis was performed using IPTG. Next, a crude lysate was obtained from the bacterial suspension (Gan et al., 2010) containing dsRNA. 2-ml aliquots of the crude lysate were prepared for treatment of N. benthamiana plants.

**Root treatment of N. benthamiana with a crude bacterial lysate containing dsRNA for silencing the pds gene**

**Fig. 3.**
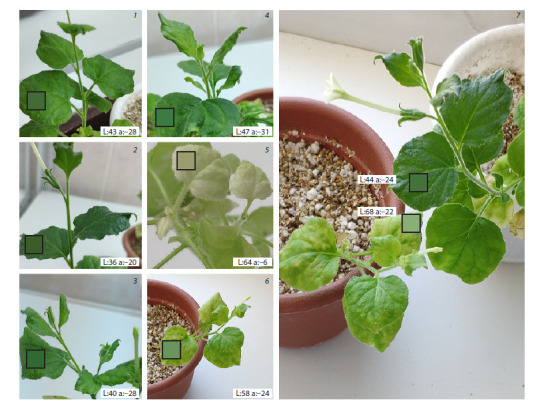
Photobleaching of N. benthamiana leaves after induced silencing of the pds gene using exogenous dsRNA. 1 – water; 2 – Tris-EDTA; 3 – E. coli HT115 crude lysate without plasmid; 4 – E. coli HT115 crude lysate with the native L4440 plasmid; 5 – VIGS; 6 – crude lysate of E. coli HT115 with the L4440 plasmid containing a fragment of the pds gene; 7 – comparison of a negative control with an experimental plant with pds silencing. Controls in the experiment: treatments 1–4 serve as negative controls, treatment 5 serves as a positive control. Characteristics of leaf photobleaching: L – lightness of color in relative units (the higher the value, the lighter the shade, from 0 to 100); a – green-red channel in relative units (the lower the value, the greener the shade, from –127 to 0).

In this study, root treatment of tobacco with a crude bacterial lysate containing exogenous dsRNA was performed for the first time. Bentham tobacco (N. benthamiana) was used as a model plant for the experiment. Experimental plants were treated with the E. coli HT115 (DE3) crude lysate with the PDS_benthamiana_L4440 plasmid. After four weeks of root treatment, N. benthamiana leaves treated with the bacterial lysate with insertion of a phytoene desaturase gene fragment showed photobleaching phenotypes of young leaves typical for pds gene silencing (Fig. 3). The leaves of N. benthamiana plants from the negative control did not change their phenotype throughout the entire treatment period.

## Discussion

Currently, there are numerous studies indicating the possibility of turning off or reducing the expression of certain genes to regulate resistance, growth, and other properties of plants by inducing RNA interference (Kamthan et al., 2015; Tiwari et al., 2017). Yet this approach involves the stage of obtaining a transgenic plant or using constructs based on attenuated plant viruses.

The problem of RNA delivery without using vector systems and modifying the genome became particularly acute after GMOs had been banned in Russia and European countries. Recently, reports have appeared in the scientific literature indicating that exogenously applied double-stranded RNA molecules (e. g. by spraying, spraying under high pressure, using RNA molecule adhesion materials, or using transfer proteins) are capable of penetrating into the plant vascular system and directly into plant cells and then inducing RNA interference, thereby increasing plant resistance to fungal and viral infections (Numata et al., 2014; Koch et al., 2016; Mitter et al., 2017; Wang et al., 2018). There are also few studies on dsRNA delivery aimed at regulating gene function by irrigation. For example, maize resistance to sugarcane mosaic virus (SCMV) infection was enhanced by this delivery method (Gan et al., 2010).

Similarly, we obtained a crude lysate containing a dsRNA fragment of the N. benthamiana phytoene desaturase gene, and carried out root treatment of tobacco for four weeks. As a result, all experimental plants exhibited the phenotype of photobleaching of young leaves, which is not uncommon for phytoene desaturase silencing. The degree of whitening was comparable to the positive VIGS control. Thus, we suggest the approach that allows regulating the activity of plant genes without creating GMOs and using only environmentally friendly methods. We believe this approach to be highly promising for implementation in agriculture in order to improve the stress tolerance of cultivated plants.

Our work is based on research by F. Tenllado and coauthors (2003), where tobacco (N. benthamiana) was successfully protected from pepper mild mottle virus (PMMoV) by spraying the above-ground parts of the plants with a crude lysate of E. coli HT115 bacteria containing dsRNA. The authors note that this method of obtaining target dsRNA molecules is quite simple and economically advantageous compared to in vitro dsRNA synthesis. The authors also show that the use of crude lysate may be more cost-effective than the use of a purified preparation, with no significant loss of efficacy observed.

Since plant protection using RNAi methods can be an alternative to the creation of GMOs, which are prohibited by law in the Russian Federation for agricultural purposes, we decided to develop the idea of F. Tenllado and co-authors (2003) and try an even simpler method of delivering dsRNA through root treatment by watering plants with crude lysate. We have shown that the introduction of exogenously synthesized dsRNA in this way can effectively influence the phenotype of plants, confirming that the introduced dsRNA enters the plants through the roots and is transported to the above-ground part of the plant, where the activity of the target pds gene is regulated.

## Conclusion

Recently, methods based on RNAi have been actively developed for plant protection, especially with the use of exogenously synthesised dsRNA, as they allow avoiding the creation of GMOs, which are prohibited by law for use in agriculture in a number of countries, including Russia. However, the use of exogenous dsRNA molecules has a number of complications. For example, the method of obtaining dsRNA must be easily scalable and cost-effective, and the delivery of the resulting molecules must be as simple and efficient as possible. In our work on dsRNA synthesis, we developed a system from the E. coli HT115 strain, transformed with the L4440 plasmid with a fragment of the Bentham tobacco phytoendesaturase gene (N. benthamiana), and for the delivery of the resulting molecules, we used root treatment – watering the plants with a coarse lysate. As a result, we were able to achieve silencing of the phytoendesaturase gene,which confirms the possibility of regulating the work of plant genes without creating GMOs. The approach we propose can also be scaled up and has potential for application in agriculture to protect plants from pathogens

## Conflict of interest

The authors declare no conflict of interest.
